# Case of Hydrophobia Successfully Treated by John Schoolbred, M. D. of the Bengal Establishment, &c. &c. First Published in the Asiatic Mirror, May 20, 1812

**Published:** 1813-01

**Authors:** 


*To the Editors of the Medical and Physical Journal.*

GENTLEMEN,

THE generally fatal consequence of hydrophobia is a sufficient apology for my calling your readers' attention to the subject, although it has of late been much discussed. The valuable remarks of Dr. Kinglake in a former number of your Journal, and of Mr. Bampfield in your last, hold out the pleasing idea that bleeding may become a likely means of obviating the destructive progress of this obstinate and cruel malady. In support of the latter gentleman's authority, you (the editors) have quoted a case from a newspaper, although you do not seem to attach implicit credence to the narrative. As it is extremely desireable that every fact, favorable to the supposition that it is possible for the complaint to be cured, should be brought before the public, I have no fear that you will refuse to admit the following narration, printed at Calcutta, which seems indeed to have been been the source whence the account of the case you published was derived. S. L.

*London, Dec.* 5, 1812.

*Case of Hydrophobia successfully treated by John Shoolbred, M.D. of the Bengal Establishment, &c. &c. first published in the Asiatic Mirror, May* 20, 1812.

Tuesday, May 5th, 1812.—About three p.m. Ameir, a Musselman *Bhestie*,* from twenty-five to thirty years of age, and middle stature, in the service of Mr. John Wood, schoolmaster, at Chowringhee, was brought to the Native Hospital, laboring under the most unequivocal symptoms of hydrophobia.

The note from Mr. Wood, requesting admission for this patient, and the friends who accompanied him, stated that he had been bitten in the leg about three weeks before, by a dog believed to be mad, and that the symptoms of his disease had appeared that morning, the 5th.

I visited him in the hospital the moment I heard of his arrival, and found him sitting on the side of a cot, with an attendant holding him by each arm. The first view was sufficient to satisfy me of the nature of his complaint. His body, arms, and throat were affected with constant and uncontrolable spasmodic startings. The muscles of his face were thrown into quick convulsive action at each inspiration, drawing back the angles of the mouth, and at the same instant depressing the lower jaw, so as to communicate the most hideous expression to the countenance. His eyes appeared starting from their sockets and suffused with blood; sometimes fixed in a wild and terrific stare, at others, rolling about as if they followed some ideal object of terror, from which he apprehended immediate danger. A viscid saliva flowed from his mouth, which was always open, except when the lips were momentarily brought together for the purpose of forcibly expelling the offensive secretion that adhered to them, and which he effected with that peculiar kind of noise which has been often compared to the barking of a dog. His temples and throat were bedewed with clammy moisture. His respiration was exceedingly hurried, and might more properly be called panting than breathing; or it still more nearly resembled that short and interrupted kind of sobbing that takes place when a person gradually descends into the cold bath. He was exceedingly impatient of restraint, and whenever he could get a hand disengaged,

* Water-carrier.

he he immediately struck the pit of his stomach with it,—pointing out that part as the seat of some undescribable uneasiness. From the constant agitation of his whole frame, and the startings of his arms, it was impossible to count his pulse with exactness; it was, however, very unequal, both in strength and frequency; at times scarcely perceptible, and then rising again under the finger; sometimes moderately slow and regular for a few pulsations, and immediately after so quick as not to be counted; but conveying, upon the whole, an idea of a greatly oppressed and impeded circulation. His skin was not hot; and, though his head was in incessant motion, accompanied with such savage expression and contorsion of countenance as might easily have alarmed those unaccustomed to such appearances, he made no attempt to bite, which is far from being a frequent symptom of the disease, and, when it does occur, must be considered merely as an act of impatience at being held, and no more than the peculiar noise above noticed, as indicating any thing of the canine nature imparted by the bite, an opinion which has been sometimes fancifully but absurdly entertained.

When questioned concerning his own feelings, or the cause of his illness, he was incapable of making any reply, being prevented, it is probable, either by the hurried state of his respiration, or by his mind being too deeply absorbed in the contemplation of horrible ideas, to admit of his attending to the queries addressed to him.

I desired water to be offered to him, at the mention of which he started with increased horror and agitation, and endeavored to disengage himself from those that held him. When one of the attendants approached with a cup of water, he looked at it wishfully, and after some efforts, with apparent reluctance, stretched out his hand to take hold of it; but before he could reach the cup, his hand was suddenly drawn back by a convulsive motion; at the same instant, he turned away his head, and writhed himself round on the bed in an agony of terror and despair, wholly inconceivable by any person who has not been a witness of the horrors of this most dreadful, and hitherto, it may be added, most irremediable of human diseases.

Such was the state of the patient at the moment of his admission, and for the few minutes that necessarily elapsed while these appearances were passing under my observation.

Of the nature of the complaint there could not exist a shadow of doubt; and, having so recently read in the Madras papers a case of hydrophobia successfully treated by Mr. Tymon, of his Majesty's 22d dragoons, by bleeding, mercury, and and opium, I determined on the immediate adoption of the same plan.

I therefore, without delay, opened a vein in the right arm by a large orifice, out of which the blood sprung with uncommon impetuosity, and of so florid a color as to resemble arterial rather than venous blood. By the time that sixteen or twenty ounces of blood had flowed, the spasmodic startings of his arms, body, and neck, had considerably diminished; his breathing had become more calm, with less contorsion of countenance; and he audibly acknowledged that the pain about the præcordia and region of the stomach was upon the decline. Encouraged by these incipient appearances of amendment, I allowed the flow of blood to continue; and, when about two pints were taken away, seeing him greatly composed, I desired water to be again offered to him, when, equally to my astonishment and delight, he took the cup in his left hand, the blood still flowing from the right arm, and calmly, but with indescribable expression of satisfaction, drank two or three ounces of water, the sight of which but a few minutes before had threwn him into the most dreadful agonies. Soon after swallowing the water, he retched three or four times, but ejected nothing but saliva from his mouth and fauces; and finding now that his pulse was 104, weak, soft, and regular; that he was become faint; and that all appearances of uneasiness had ceased, so as to allow him to take a second draught of water, about four ounces, I closed the vein and laid him down on the bed. At this moment he expressed a desire to have a natural alvine evacuation, and wished to go out of the hospital for that purpose; but, as that could not be complied with, he took no more notice of it at this time. It is worthy of remark also, that during the bleeding he made a sign to have himself fanned, a thing I never knew a patient in hydrophobia do before, their distress being so uniformly increased by any current of air blowing upon them, that, according to all my experience, the dread of air in motion is as constant an attendant on the disease as the dread of water itself.

After the bleeding he remained perfectly quiet, and fell into a slumber for about an hour. Another circumstance which also strongly marks the abolition of the disease, as no hydrophobic patient was ever known to sleep. When he awoke, he expressed a wish to have some sherbet, which was immediately given to him, and he drank four ounces of it with perfect ease. He then fell into another slumber, during which some convulsive startings were again perceptible about his arms, chest, and face, but not strong enough to wake him.

At a quarter past five he spontaneously awoke, and appeared again somewhat agitated, with more suspicion in his looks, and of apparent doubt whether he could swallow as well as before; for, when he took the cup, he put it to his lips with a quick motion, and gulped down about four ounces of water in a hurried manner, as if afraid that the difficulty of swallowing would be increased by a moment's delay. He also put his hand to the region of the stomach, and said that the pain in that part was returning. These threatening appearances of relapse determined me to hazard a farther detraction of blood. I therefore immediately opened a vein in the left arm, and allowed the blood to flow again till he completely fainted; but previous to this effect of the bleeding, the pain at the stomach had ceased, and while the blood was yet flowing, he had again drank four ounces of water without fear or disgust. When he recovered from the fainting fit, he retched several times, but, as before, discharged nothing but saliva.

At the end of the first bleeding his pulse was 104; immediately before the second it was 96, with a slight degree of sharpness in the beat; and, after recovering from the fainting occasioned by the second bleeding, it was 88, regular, soft, and feeble, and he now complained of nothing but extreme weakness, and giddiness of the head. And at this stage of the case, I apprehend, it will be allowed that the cure of the hydrophobia was complete—whether it would be permanent or not remained yet to be seen.

When I began the treatment of this patient, it was my intention, as I have said, to follow in every circumstance the practice pursued in Mr. Tymon's successful case; and accordingly a draught with 100 drops of tincture of opium, and an enema of 300, were in readiness to be administered immediately after the bleeding. But, seeing the surprising effects of the bleeding alone, and feeling convinced that the disease was, for the present at least, completely annihilated by the copiousness of that evacuation, I determined to preserve the treatment as simple as possible, in order that if the patient did finally recover, it might with certainty be known to what he owed his safety; and that thence the application of the same practice to future cases of hydrophobia, might with the greater confidence be recommended:—a resolution in which I was the more confirmed from having heard some medical friends, whose opinions are entitled to every degree of respect, ascribe Mr. Tymon's success to the mercury he had used, rather than to the bleeding.

I am now fully persuaded, however, that I might safely, as far as the hydrophobia was concerned, have omitted all remedies after the bleeding; but, thinking that calomel and

 opium, opium, in repeated doses, were more likely than any thing else to induce that state of the system which would be least favorable to a relapse; and also that, if the patient, notwithstanding his present promising appearance, did not finally recover, it would certainly be said that I had not given him a fair chance, by departing in any particular from the treatment which had proved so successful in the hands of Mr. Tymon, I was led to conform to it so far as to order four grains of calomel and one grain of opium to be given every three hours.

The first pill was taken at a quarter before six, but it was immediately rejected, followed by some water. A second was given five minutes before six, and remained. He now slept till seven, then drank some more water, and had a natural evacuation of his bowels—another circumstance which confirmed me in the belief that the disease was completely and permanently subdued, having never before seen it, nor read in any history of the disease, of such an occurrence as a natural action of the alimentary canal, in a case of hydrophobia.

At nine he took another pill, and again at twelve, and continued to slumber and drink water as often as he pleased.

Wednesday, May 6th, (second day) six a. m.—Has passed the night well. Took a pill at three, and another now. Has drunk water frequently. Pulse 84. Skin cool. Tongue clean at the edges; some remains of beetle, eaten before he was taken ill, cover the centre part. Two more alvine evacuations during the night. Complains of head-ach, but is entirely free from uneasiness about the stomach.

On examining the blood drawn yesterday, the surface of the coagulum is found not to be in the least concave, neither does it exhibit the slightest appearance of what is called the buffy coat. The quantity first drawn, making allowance for the evaporation of the night, measures forty ounces; and the last between seven and eight.

Nine a. m.—Took another pill, which was followed by another evacuation; and in half an hour afterwards, he ate eight ounces of sago. Is quite composed, and can answer questions distinctly concerning the accident and subsequent occurrences, till the time he was taken ill.

He says that nineteen days ago (including this day) when returning about four in the afternoon from his own house at Russapuglah, to his master's at Chouringhee, he saw a pariah dog seize a fisherman, and bite him. Several people were collected at the spot: he also approached, when the same dog ran at him, and, as he was retreating before him, bit him in the back part of the right leg, about six inches above above the ankle, where he shews two scars at the distance of an inch and a half from each other, but without any appearance of inflammation or thickening of the integuments. The dog, after biting him, disappeared, and he does not know what became of him or of the fisherman. The wounds bled a good deal, but not being very deep, they soon healed, without any application. He took no remedy, except, on the day he was bitten, a small piece of scarlet cloth *(sooltanee banat)* wrapt up in a piece of ripe plantain, which was recommended to him as an infallible antidote against infection from the bite of a mad dog. He never saw any one in hydrophobia, and, though he had heard that persons bitten by a mad dog were liable to such a disease, the apprehension of it never dwelt on his mind, or scarcely ever occurred to him after the day on which he was bitten. He continued in his usual health till the 4th instant, seventeen days after the bite, when he found himself dull, heavy, and listless, with loss of appetite and frequent apprehension that dogs, cats, and jackalls, were about to seize upon him. He also felt a pricking sensation in the part bitten. When his mother-in-law brought him his breakfast, he was afraid to eat it. He continued his business, however, of taking water from the tank to the house, till about noon of that day, after which he could not bear to look on or to touch the water, being constantly harassed, whenever he attempted to do so, with the horrible appearance of different animals ready to devour him. He now, for the first time, thought of the disease arising from the bite of a mad dog, was convinced that that was the cause of his present distress, and fully believed he should die of it. He ate no supper, nor drank any water that night, in consequence of the horrible phantoms that incessantly haunted his imagination. In the morning, all his horrors were increased, the spasms came on, accompanied by anxiety, oppression, and pain about the præcordia and stomach; and those about him say that he continued to get worse in every respect, until he arrived at the hospital in the state already described. He does not himself distinctly remember any thing that happened during the whole day. He has some faint recollection of having been at his own house; but how he got there, when he left it, or by what means he was brought to the hospital, he does not at all know. The first thing he can rec[illegible] to his mind is drinking the sherbet; and he says, he has had his senses perfectly since that time, and that all his fears then left him, and have not since returned. This, however, is not entirely correct, as he acknowledges that he does not recollect the second bleeding, bleeding, which shews that the disease had then so far returned as again to disorder his mental faculties.

Half-past ten a. m.—Complains of severe head-ach, and his eyes are more suffused than they were in the morning. No return of other symptoms.

Head shaved, and six leeches applied to each temple.

Three p. m.—Took a pill at twelve, and another just now. Leeches bled freely. Head-ach relieved. Took eight ounces more of sago about noon.

Six p. m.—The same. Has now taken twenty-eight grains of calomel and seven of opium. To take from this time only two grains of calomel and half a grain of opium every three hours.

Nine p. m.—Has slept for two hours. Pulse 80. Took another of the pills last ordered, also some more sago. Copious bilious evacuation. Still complains of giddiness, but not head-ach.

Thursday, the 7th, (third day) six a. m.—Took a pill at twelve, but refused one at three, saying his mouth was sore. Took one now. Has been rather restless in the night. Threw up some bile this morning.

Ten a. m.—Exceedingly distressed by excessive secretion of bile, which he is frequently throwing up and also passing downwards in great quantity; and of a dark green color. Pulse 110. Some heat of skin; expression of uneasiness in his countenance; burning sensation all over the abdomen, but quite different, he says, from the former pain about the stomach. He was ordered a pint of infusion of camomile, which brought off much bile. At eleven, eight grains of calomel; and, at half past twelve, half a dram each of jalap and magnesia. By the effects of these remedies, he was much relieved in the evening, though the complaint continued to disturb him in the night, and it was necessary on

Friday morning the 8th (fourth day), to promote the farther evacuation of bile by senna, manna, and cream of tartar; and to order an enema of conjee to allay local irritation. Pulse only 80, soft. Burning removed from the abdomen. Ate a water melon in the night. Copious flow of saliva from his mouth.

Saturday, 9th (fifth day), nine a. m.—Has passed a good night. Excessive secretion of bile has ceased. Clamorous for food, but I allow him only rice and sago; declines milk. He appears now to be free from all complaint. After this time nothing remarkable occurred. He had a strong appetite, and was allowed vegetable curry. For several evenings some heat of skin and acceleration of pulse were perceptible; but but these soon went off from cold bathing, and a constant attention to keep his bowels in an open state.

Monday, May 18th (fourteenth day).—Has been for some days past on the usual hospital diet, and, feeling himself well in every respect, now expresses a wish to be discharged and return to his ordinary business; but, as the weather is exceedingly hot (thermometer in the shade from 95° to 100°), I have prevailed upon him to continue in the hospital till the setting in of the rains. I shall then, if possible, persuade him to remain in my own employment for the next twelve months; lest, if he were discharged, and should happen to die of whatever disease, it might be alleged that he was after all carried off by a relapse of the hydrophobia.

*Remarks.*—On hearing that a recovery from hydrophobia has been effected in the short space of two hours, by the single remedy of blood-letting, a doubt may probably occur to a person acquainted with the previous history of this formidable malady, and the nearly uniform failure of all attempts, hitherto made for its cure; whether the disease now said to be cured, was in reality a genuine case of hydrophobia, produced by the bite of a rabid animal. I admit the scepticism to be reasonable; for, in the relation of a case, which has terminated so differently from all others yet on record, (not even excepting the case so successfully treated by Mr. Tymon,) it is natural to suspect either some misconception or misrepresentation of facts; or some fallacy in the deductions derived from them.

An attentive perusal of the preceding narrative will, it is presumed, remove these doubts from the minds of the majority of readers: yet, as some individuals may not be convinced by that evidence, which to others appears full and satisfactory; and as it is a matter of the utmost importance to future sufferers from hydrophobia, as will be more fully shewn hereafter, that no doubt should be allowed to remain, either as to the existence of the disease itself, in the case above related, or that the bleeding was the sole remedy; I shall, as briefly as possible, endeavor to establish the certainty of both those facts, beyond the possibility of contradiction.

To a person who has never seen a case of hydrophobia, I acknowledge the difficulty, nay, almost the impossibility, of conveying by words an adequate notion of the disease. The horrors of that state must be seen to be fully conceived; but, being once seen by a medical observer of any discernment, they are indelibly fixed in the mind; and I contend that it would then be highly improbable that he should ever mistake any other disease for hydrophobia; or take hydrophobia for for any of those affections to which it has been said to bear some resemblance;—so deep and so permanent, I am convinced, would be the impression left on his mind by the contemplation of even a single case of hydrophobia. But when I state that my situation as surgeon to the Calcutta Native Hospital, for the last eighteen years, has afforded me opportunities of seeing the disease, which have fallen to the lot of few individuals in any country, and that no less than seventeen or eighteen cases of it have come under my observation within that period, in all of which both my diagnosis and prognosis (with the single exception of the latter in the case under consideration) have unhappily been but too fatally verified, it is not, I trust, laying claim to too great a share of discernment to assert that I could not easily be mistaken in a case of hydrophobia; and that I should consider my being so as unlikely, as that an experienced surgeon should ever confound two diseases the most opposite in their nature; because, to an uninformed eye, they might both exhibit something of the same external appearance.

Farther; it has been usual with me, on the admission of a case of hydrophobia into the hospital, to send for some of my medical friends, not only that they might see a disease seldom occurring in private practice, but that I might have the benefit of their suggestions in regard to the treatment. On the present occasion, the promptitude necessary to the practice I had determined to adopt in the first case that occurred; and its astonishing effect in so suddenly and effectually subduing the disease, deprived me of the advantage I should now have derived in establishing the point in question, from the concurring testimony of a judicious medical friend. But, though not permitted to give direct evidence as to the existence of the disease in the case above detailed, these gentlemen can yet vouch, that they were never called by me to see a case of hydrophobia in which there existed the slightest doubt of the nature of the disease; and it will hardly be contended that I was more liable to mistake it in this case, than on any former occasion.

If these facts and reasonings, combined with the account of the accident;—the time that elapsed before the appearance of the symptoms;—the statement given by the patient of the commencement of the disease;—and by his friends, as to the state in which he appeared before he was brought to the hospital;—and the symptoms under which he labored when he arrived there;—should all be deemed insufficient to establish the real nature of the disease, I confess myself at a loss to conjecture what species of proof would be necessary for that purpose. The only defective point in the evidence dence appears to be our ignorance whether the dog by which Ameir was bitten, was actually mad or not? And, though this cannot be proved by direct testimony, yet as it is known that the disease was prevalent among dogs, about that time, as will be hereafter noticed, it is presumed that this is an objection of very little weight. If therefore any individual, after duly considering all these circumstances, still continue in doubt as to the nature of the disease, may it not in conclusion be permitted to ask him what disease it was, if not hydrophobia?

That the disease, whatever it might be, was removed, and that almost instantaneously, by bleeding alone, admits, in my mind, of equally little doubt.

In Mr. Tymon's successful case, the symptoms only gradually disappeared, some of them remaining so late as the fourth day; and, as opium, mercury, and antimony, had been largely used during the whole time, and the patient's system was evidently under the influence of the mercury, before he could be said to be free from the disease, an opinion might still be entertained, and actually was so, by many, with whom I have conversed on the subject, that the cure was, after all, effected by the mercury, and not by the bleeding.

Dr. Berry himself, to whose rare and laudable zeal for the promotion of useful science, even at the period of closing a long and honorable career of public service, the world is indebted for the knowledge of Mr. Tymon's unprecedented case of success, alleges, that the bleeding "saved Mason's life by diminishing violent action, *and admitting the effect of medicines that in all former experience had uniformly failed.*"

As this notion too corresponds with the most prevailing theory of the disease—though that theory has not in a single instance been verified by the success of the practice to which it gave rise, I consider it of great importance to correct it; lest, by still expecting some good from mercury and opium in hydrophobia, the attention of the physician should be diverted from a sufficient abstraction of blood,—on which, and on which alone, as far as a single case can prove any thing, the life of the patient seems entirely to depend.

That the first bleeding in the case above related wholly, though not permanently, removed every symptom of the disease, was proved, I presume, in the most ample manner, by the following six remarkable circumstances: 1st, the removal of the spasms; 2d, the freedom of respiration; 3d, the restoration of the power of swallowing fluids, and the absence of horror at their approach; 4th, the desire, instead of of the abhorrence, of a current of air; 5th, the inclination for a natural alvine evacuation; and, 6th, the power of sleeping. All these unequivocal indications of recovery took place during or immediately after the first bleeding; and, as none of them ever happened before to a patient in hydrophobia, except near the close of the melancholy scene, when they denote an entire sinking of the powers of life, rather than the cessation of disease, it seems but fair to ascribe them to a remedy, which had never before been used as it was on this occasion; or, if so, unluckily not at the time when it was capable of doing good.

When a recurrence of the disease was threatened in two hours afterwards, the power of the remedy was again conspicuously manifested, and a second bleeding *ad deliquium* instantly stopped the progress of the symptoms, and, before a single particle of medicine of any kind had been given, permanently extinguished the morbid condition, whatever it may be, in which the essence of the disease consists.

These two points, therefore, appear to be fully proved, namely, that the disease was hydrophobia, and that the cure consisted in blood-letting alone.

But, notwithstanding this unprecedented success, I am not so sanguine as to believe that venesection will cure every case of hydrophobia. It is probable that there is a period in the disease beyond which its curative effect cannot extend. What that period is, cannot be known without a more enlarged experience. But this very uncertainty affords only a more powerful reason for losing no time, in resorting to the copious abstraction of blood, upon the very first appearance of unequivocal symptoms of the disease, as the delay of only a few hours may prove fatal to the patient.

In referring to notes which I have preserved of fourteen cases of hydrophobia, I find that eight of the patients died within six hours after admission. In these I cannot believe that bleeding would have done any good. But of the remaining six, who lived respectively 11, 13, 15, 20, 36, and 49, hours after admission, it is certainly reasonable to believe that it might have saved three or four. In a case so entirely hopeless, however, there could scarcely be harm to the individual, from trying it at any period of the disease. And, as it is only by such trials that the real limits of its power can ever be ascertained to any useful purpose, it is rather desirable than otherwise, that they should be made. One disadvantage, however, eventually arising from such trials, requires to be guarded against. The medical profession, taught by innumerable disappointments, admit very cautiously the claims of any new mode of practice to general adoption.

adoption. If several patients in hydrophobia, therefore, should happen to be bled in an advanced stage of the disease, and die,—as they inevitably would do, whether they had been bled or not,—such cases would be quoted against the new practice as failures, and might tend so far to bring the remedy into discredit, as to prevent its being used, even in cases where it might have proved the certain means of saving life.

I am the more desirous of noticing the unfavorable effect upon the adoption of the new practice, which may eventually arise from bleeding at too late a period of the disease, and of entering a strong caution against the hasty rejection of the remedy from such instances of failure, in consequence of the circumstance having very nearly happened to myself, only three days before the occurrence of the case of Ameir.

On Saturday evening, the 2d of May, 1812, a native of Arracan, employed in Calcutta as a cook, was brought to the hospital, laboring under symptoms of hydrophobia. I went to him that moment, with the full determination of putting in practice the plan that had succeeded in the hands of Mr. Tymon; but I found that the unfortunate sufferer had been ill, according to the account of his friends, for 56 hours. His pulse was imperceptible, his skin cold, and his features sunk. I therefore got him to swallow 100 drops of laudanum, which he effected, as frequently happens, with greater ease than is usual in an earlier stage of the disease; and ordered an enema, with 300 drops. The patient was dead in half an hour. Now, what I wish to impress upon the mind of the reader is, that, if, in this case, the disease had been somewhat less advanced, the pulse still perceptible, and the strength less sunk, I should certainly have bled the patient; which at such a period could scarcely have prevented death. It would more probably have appeared to have accelerated that event; and, if so, might consequently have had the effect of preventing my pushing the bleeding in the case of Ameir, to the extent necessary to the cure. I must therefore here insist, that numerous failures in an advanced stage of the disease, will form no just ground for the rejection of a remedy, which has been so incontestably proved to have cured the disease when used at an earlier period. As well might the practitioner reject bleeding in the commencement of peripneumony or enteritis, in a robust athletic patient, because in each disease there is a period after which the detraction of blood, so far from curing, serves only to hasten the fatal event.

Nothing, however, can fix the real value of the remedy but experience. It is highly desirable that this may be speedily obtained; and, as the disease does, and must very frequently, occur in this country, whether we possess the means of curing it or not, we cannot doubt but that a very short time will elapse without further trials of this practice; and it may be presumed that the medical practitioners, who are so widely distributed throughout India, will fairly and circumstantially communicate to the public, the result of their experience, whether attended with success or not.

It may be necessary to observe, however, that merely opening a vein, and drawing a considerable quantity of blood, is *not* the practice. The vein must be opened by a large orifice, the blood quickly evacuated, and allowed to flow, without regard to measurement, ad animi deliquium. Nothing less than this is capable of at once arresting the progress of the disease, relieving the spasmodic affection of the heart and arteries, suppressing excessive sensibility and irritability, and, in short, of admitting the restoration of that due balance of action and influence both in the circulating and nervous systems, on which the continuance of life and health seems to depend.

But I lay no stress on this or any other pathology of the disease. Well authenticated trials of the remedy in an early stage of it, are what I desire to see. If it fails in many of these, when used in the manner above proposed, within twenty-four, or, to speak with more latitude, thirty, hours, of the commencement of the symptoms, I confess I shall feel much disappointed; and not a little mortified, to be obliged, after such fair prospects, to reject a remedy, which has effected twice, in the short space of seven months, what was scarcely ever effected before; and to class it with that useless farrago of remedies and practices, which, though used hundreds of times, and for a series of ages, have never once been satisfactorily proved to accomplish a cure of hydrophobia.

With respect to the subsequent treatment of the patient, it is scarcely necessary to make any remark. The case clearly shows that for the hydrophobia no subsequent treatment was required. But as this, and many other cases on record, shew a great disposition to disordered and excessive action of the liver, it may perhaps hereafter be found useful to administer mercury both as an evacuant, and to the extent of affecting the mouth, with or without opium, according to circumstances.

It is usual when new and successful expedients are first promulgated, to wonder why they never were thought of before. In conformity to this habit, I have frequently, within the last ten days, been asked, why in a disease so often often proved incurable by other means, bleeding was not before tried?

The fact is, however, that *bleeding has often been tried.* But, owing, probably, to the evacuation not being pushed far enough, when used in an early stage of the disease, or to the period for its beneficial employment having elapsed, before it was resorted to, the relation of the cases in which it was used afforded little or no encouragement to farther trials; while the theory that has prevailed for nearly a century in regard to the nature of the affection; and its classification with diseases of the nervous kind, accompanied by great debility, tended directly to discourage all lowering plans of cure, and to point out antispasmodics and tonics as the only resource in hydrophobia.

Dr. Mead, who was very confident that he had found an infallible preventive of the disease, in *a little liverwort and black pepper*, aided by bleeding and cold bathing before the commencement of the course of medicine, says, "as to all other ways of curing the hydrophobia, I own I have not been so happy as to find any success from the many I have tried. Bathing at this time is ineffectual. *I have taken away large quantities of blood;* have given opiates, volatile salts, &c. &c. &c. All has been in vain, *because too late.*" Notwithstanding his disappointment, he still concludes, "if any relief could be expected in this desperate state, I think it would be from *large bleeding, even ad animi deliquium, before the fibres of the membranes have lost their natural force by convulsions.*" But, after all, it will generally happen, that (as the Greeks said upon deplorable cases) "Death will be the physician that cures." This, though a recommendation, was certainly no great encouragement to blood-letting.

The doctrines of Boerhaave also led him and his pupils to recommend and practice bleeding in hydrophobia. The celebrated Leyden professor says, "the distemper is to be treated as one highly inflammatory, upon the first appearance of the signs which denote its invasion, by blood-letting from a large orifice, continued till the patient faints away; and soon after by enemata of warm water and vinegar," &c. &c. and he adds, "that this practice is supported by some small number of trials." But the particulars of this successful practice are not given.

I find, however, a trial of it at Edinburgh, more than sixty years ago, by the late Dr. Rutherford, a pupil of Boerhaave's, who took away *gradually* sixty-six ounces of blood from a patient, who had already been bled the same morning. As this patient lived forty-eight hours after the large bleeding,

 it it is probable that it was used somewhat early in the disease, and should, therefore, it may be said, have succeeded. Why it did not, it is impossible now to tell, unless the word *gradually* may be thought to afford some explanation; but I am persuaded the circumstances attending its failure had great weight in deterring others from pursuing the plan recommended by Boerhaave, and in giving an entirely different direction to the practical views of physicians on the subject of hydrophobia.

On the failure of bleeding in this case, Dr. Rutherford, who then, with great reputation, filled the practical chair of the most celebrated school of medicine in Europe, candidly retracted an opinion, which he had learned from Boerhaave, and which had directed the measures he took. He declared in his public lectures, that "he was convinced now that the hydrophobia is a spasmodic and not a high inflammatory disease. That, though bleeding may be useful in preventing furiousness, neither that, nor the proper antiphlogistic method, are to be depended upon as the proper cure of hydrophobia; that in such cases, after bleeding once or twice, he would order *sal succini*, *musk*, *opium*, and perhaps *blisters*." Thus at once sending abroad into all parts of the world the opinion that large bleeding was useless in hydrophobia, and inculcating the use of antispasmodics only.

Dr. Cullen says scarcely any thing on hydrophobia, farther than that his chief reliance would be on mercury.

Macbride asserts that "Dr. Nugent was the first that pointed out the true nature of hydrophobia; which, before his time, was generally considered as an inflammatory disease. Dr. Nugent's patient was *largely blooded*, and took, moreover, large quantities of musk and cinnaber as well as opium; and, toward the close of the cure, opium was given along with camphor, musk, and assafœtida. *But the opium is what we are chiefly to rely on.*" Thus again withdrawing the attention of the practitioner from the large abstraction of blood, to which the cure in this case was most probably to be ascribed.

It is needless to multiply quotations, to prove that nearly the same opinion of the disease, and the remedies most applicable to it, have prevailed with little variation up to this day, with the single exception, perhaps, of Dr. Rush, who, in consequence of his peculiar notions about inflammation, but which do not seem to be countenanced by the appearance of the blood drawn from hydrophobic patients, again inculcated the necessity of blood-letting. Recent experience proves that he was right. But it is to be regretted that neither the cases to which he refers for the success of the the practice, nor his amended hypothesis of the resemblance of hydrophobia to malignant fever, were considered of sufficient weight to encourage its adoption by other practitioners.

Finding, therefore, so many authorities against bleeding in hydrophobia, and not a single cure fairly ascribed to it (for no dependance can be placed on those vaguely mentioned by Boerhaave), it is by no means surprising that it should, for more than half a century, scarcely ever have been thought of as a remedy in this disease. I am aware that it has sometimes been used as an auxiliary when the pulse has been full and the strength great, in order to render the patient more manageable. But, as it has till lately never been employed as the remedy of sole dependence, nor applied in the manner necessary to produce a decided effect upon the disease, I confidently trust that its failure nearly up to the present day will not be considered as militating against the expectation of success which I think we are now fairly entitled to entertain from its future employment.

It is, at any rate, highly encouraging to know, that, in the only three cases in which it has been trusted to as the principal, or the sole, remedy, it has succeeded to our utmost wishes.

The first case is that by Dr. Burton, in America, which was suggested by Dr. Rush's lectures; and was published about seven years ago, in different periodical works. But unfortunately, in consequence of the case not being very accurately related, and its being combined with some fanciful theory, it does not appear to have been acknowledged as a clear instance of hydrophobia; and the benefit which might otherwise have been derived from it, was wholly lost to the world. Whether it was actually a case of hydrophobia or not, is not now worth disputing, being in possession of Mr. Tymon's case, and of that which has given rise to these already too greatly extended remarks.

I cannot, however, conclude without saying a few words on the practices which have been principally in use up to this time.

Never having seen Dr. Nugent's case, the only instance of apparently authenticated recovery from hydrophobia with which I was acquainted, previous to these three, is one related by Dr. Shadwell, in the Memoirs of the London Medical Society, in which, on the authority of a Greek manuscript, oil was used both externally and internally. Relying on this solitary example of success, I gave oil a very fair trial in several of the first cases that fell under my care. But, although I often got the patient to swallow a considerable quantity quantity of it, and applied it frequently by enema, as well as to the skin, by almost incessant frictions, it never appeared to do the least good. I therefore abandoned it.

I have subsequently used every mode of treatment that I have ever heard or seen suggested, with equally little success, except arsenic, which, though with no better hope, was to have been my next trial, had not Mr. Tymon's case fortunately occurred, to point out the practice which has already so well justified the confidence reposed in it.

On those occasions, besides the full trial given to oil, I used opium to a great extent in every possible way; mercury, musk, camphor, blisters, galvanism, and enemata of laudanum and infusion of tobacco, all to no purpose. Nothing ever alleviated a symptom except the two last, which certainly did lessen the spasms; and, therefore, when bleeding may hereafter be used too late to succeed, I would recommend them as remedies, capable, though not of preventing death, yet of allowing the fatal event to take place with less suffering to the unhappy patient than any thing else with which I am acquainted.

On the recommendation of Dr. Bardsley, of Manchester, a gentleman who has, with unwearied zeal, endeavored to investigate the nature of hydrophobia, with a view to the discovery of its cure, and even to the extermination of the disease from the United Kingdom, I also gave a very fair trial to volatile alkali. Contrary to all expectation, I succeeded in getting into the stomach no less than three drams of carbonate of ammonia, made into bolusses with crumb of bread. But the event was unhappily just the same as in all former cases.

Dr. Bardsley was led to this suggestion by the perusal of Mr. Williams's cases of recovery from the bite of the *Cobra de Capello*, by means of *Eau-de-luce;* and he endeavors to recommend its adoption by the following observation: "surely, in the treatment of so fatal a disease as canine madness, it is proper to adopt any method of cure founded on RATIONAL PRINCIPLES. *Analogy, under these circumstances, seems to be our surest guide.*"

It is melancholy to relate, that, though hydrophobia has been unusually frequent in England of late years, and many cases of it have been treated by the most eminent practitioners in London, both in hospitals and private practice, yet not a single case of recovery is recorded.

Dr. Parr, author of the Medical Dictionary, published for the express purpose of exhibiting the state of medical science up to the present time, after telling that every thing has been tried, and that every thing has failed in effecting a cure, cure, consoles his reader by acquainting him with the infallibility of cutting out the part as a preventive, adding emphatically, in italics, "*In short, full, effectual, and* COMPLETE, EXCISION *of the wounded part, is the only certain means of relief;* AND THIS IS CERTAIN." But still leaving us in the same hopeless condition as to any means of cure after the disease has actually taken place.

Dr. John Hunter concludes a most able paper on the history of the disease, and the trials made for its cure, with these words: "after the symptoms of hydrophobia have appeared, no medicine or remedy that has hitherto been used, has relieved, much less cured, the disease;" and, finally,

A well-informed anonymous writer, in the Medical Annual Register for 1808, after presenting a sketch of the practice that had been pursued in London during that year, and noticing the failure of every expedient, sums up his history with this opprobrious sentence: "On the whole, therefore, we may be considered as remaining in the most entire ignorance both of the nature of the disease, and of the method of cure, or even of palliating a single symptom."

Such was the disheartening language universally held on the subject of hydrophobia. I humbly trust it can be held no longer; that the case above related, coming so soon after that of Mr. Tymon, entitles us to indulge more animating views for the future; and that it will not be long before additional experience shall serve to confirm the hope which seems now to rest on so promising a foundation,—that a remedy has at length been discovered for this hitherto uncontrolable disease.

It is mortifying to the pride of science to acknowledge it, but, if farther trials of bleeding *ad deliquium* shall confirm its power of curing hydrophobia when used early in the disease, it is nevertheless impossible to conceal that this *solum et unicum remedium*, has apparently been hitherto overlooked in consequence of an overweening fondness for system, which led medical writers to class hydrophobia with diseases of the nervous kind, and to dwell particularly on its resemblance to tetanus. That disease being considered as highly asthenic, blood-letting, perhaps without sufficient reason, has been thought inadmissible. Antispasmodics and tonics have been employed in all quantities and forms, and though, by such remedies, scarcely one case of tetanus in fifty has ever been cured, the same treatment has been rather preposterously it should seem, transferred to hydrophobia, because, under such hopeless circumstances, *analogy has been said to be our surest guide.* Whither has it guided us? Never certainly to a single cure of hydrophobia: it may, perhaps, perhaps, with greater truth be said to have been an *ignis fatuus*, serving to lead us into difficulties and dangers, rather than to conduct us into the salutary path of curative science; and that, perhaps, in more diseases than the one under immediate consideration.

After expressing so little respect for analogy, the professed guide of physicians, in the treatment of hydrophobia, shall I not be accused of inconsistency, or of indulging in notions of too speculative a nature, if I offer a hint that some use may yet be derived from this favorite doctrine, by pursuing the analogy in an opposite direction; and, instead of applying to hydrophobia the treatment which seldom succeeded even in tetanus itself, we now transfer to tetanus, and, perhaps, to other diseases of the same kind, the practice which has been incontestibly proved, in two instances at least, if not in three, to have been successfully employed in hydrophobia?

Almost all authors have spoken of this analogy, and some have gone so far as to affirm, that tetanus may be easily mistaken for hydrophobia. I confess myself to be of a different opinion, being fully persuaded that no person who has often seen both diseases, could ever mistake the one for the other; and that for the following reasons:—first, in tetanus the lower jaw is immoveably fixed, and the patient speaks by the motion of his lips only, with a hissing kind of noise; whereas, in hydrophobia, the lower jaw is moveable to any degree, and is, in fact, in the exacerbâtions, almost in perpetual motion, often resembling the action of hawking or retching, for the purpose of bringing forward and expelling the viscid saliva, which constantly collects about the fauces: and, in the second place, that, though the swallowing of fluids may be difficult or impossible in tetanus, and the attempt even accompanied with convulsions of the face, throat, and chest, yet the obstacle is confined to the actions connected with deglutition alone; and the name, the sight, the approach, and the touch, of fluids, have never, in my experience, thrown the patient into the agony of horror, distress, and despair, which is invariably witnessed in hydrophobia.

